# Prevalence of Anxiety and Depressive Symptoms in Patients With a Positive Hoffmann Sign and No Cervical Cord Compression on MRI: A Cross‐Sectional Study

**DOI:** 10.1002/hsr2.72988

**Published:** 2026-08-10

**Authors:** Ebrahim Hejazian, Forouzan Sedaghat, Sussan Moudi, Hoda Shirafkan, Hadi Ebrahimi, Hamed Ahmadian, Amir‐Hosein Zohrevand, Mostafa Esmaeilnia Shirvani, Morteza Sharifzadeh, Amirreza Tahmasebzadeh

**Affiliations:** ^1^ Department of Neurosurgery Babol University of Medical Sciences Babol Iran; ^2^ Student Research Committee Babol University of Medical Sciences Babol Iran; ^3^ Social Determinants of Health Research Center, Health Research Institute Babol University of Medical Sciences Babol Iran; ^4^ Department of Neurosurgery Beheshti Hospital Mazandaran Iran; ^5^ Babol University of Medical Sciences Babol Iran

**Keywords:** anxiety, cervical myelopathy, cross‐sectional study, depression, Hoffmann sign, hospital anxiety and depression scale

## Abstract

**Background and Aims:**

Hoffmann sign is commonly used during screening for cervical myelopathy, but its diagnostic performance varies across studies. Psychological factors such as anxiety have been proposed as possible contributors to false‐positive or variable reflex findings, yet data are limited. This study aimed to describe the prevalence of anxiety and depressive symptoms among patients with a positive Hoffmann sign but no cervical cord compression on magnetic resonance imaging (MRI).

**Methods:**

This descriptive cross‐sectional study included 99 consecutive adults evaluated in a neurosurgery clinic who had a positive Hoffmann sign and underwent cervical MRI as part of clinical workup. Patients with significant cervical cord compression or T2 cord signal change were excluded. Anxiety and depressive symptoms were measured using the Hospital Anxiety and Depression Scale (HADS). Results are reported as counts, percentages, means with standard deviations (SDs), and 95% confidence intervals (CIs) where appropriate. Associations with age group, sex, and history of psychiatric consultation were exploratory.

**Results:**

The cohort included 99 participants; 89 were female (89.9%; 95% CI, 82.4%–94.4%). Clinically significant anxiety symptoms (HADS‐A > = 11) were present in 51 of 99 participants (51.5%; 95% CI, 41.8%–61.1%), and clinically significant depressive symptoms (HADS‐D > = 11) were present in 40 of 99 participants (40.4%; 95% CI, 31.3%–50.3%). Borderline or clinically significant symptoms (HADS > = 8) were observed in 89 of 99 participants (89.9%) for anxiety and 80 of 99 participants (80.8%) for depression. The history of psychiatric consultation was associated with higher anxiety and depression categories in exploratory analyses.

**Conclusions:**

In this selected clinical cohort, anxiety and depressive symptoms were common among patients with a positive Hoffmann sign and no MRI evidence of cervical myelopathy. These findings support cautious interpretation of an isolated positive Hoffmann sign and justify further controlled studies. The results should not be interpreted as proving causality or as a basis for limiting neurological workup when objective upper motor neuron signs are present.

## 1 Introduction

Cervical myelopathy (CM) is a clinical syndrome caused by compression of the cervical spinal cord, most commonly from degenerative changes such as cervical spondylosis or disc herniation. It may present with hand clumsiness, gait disturbance, weakness, sensory symptoms, hyperreflexia, and other upper motor neuron signs. Early recognition is important because delayed diagnosis may lead to irreversible neurological impairment, and treatment is often directed toward preventing further neurological decline [1, 2, 3].

Magnetic resonance imaging (MRI) is the principal imaging modality used to evaluate suspected CM, with relevant findings including cervical cord compression, narrowing or obliteration of the subarachnoid space, and intramedullary T2 signal change [4, 5]. However, physical examination remains central to identifying patients who need further diagnostic evaluation and to interpreting imaging results in their clinical context [5].

Hoffmann sign is one of the most widely used upper‐limb reflex signs in neurosurgical and spine practice. It is elicited by flicking the distal phalanx of the middle finger and observing for involuntary flexion of the ipsilateral thumb and/or index finger [3, 6]. The sign was described in the early 20th century and has traditionally been regarded as a marker of corticospinal tract dysfunction [6, 7, 8, 9].

Despite its frequent use, the diagnostic performance of Hoffmann sign for CM is variable [10, 11]. Reported sensitivity ranges from 20% to 89%, and reported specificity ranges from 36% to 100% [12, 13]. Some authors have considered the sign useful as part of a broader screening examination, whereas others have concluded that Hoffmann sign alone cannot confirm or exclude CM [3, 6, 12, 13].

This variability may reflect differences in patient selection, disease severity, examiner technique, test definition, and non‐spinal contributors to reflex excitability [14]. Prior work has considered technical factors such as wrist position and anatomical factors such as the level of cord involvement [15]. Psychological arousal has also been suggested as a possible influence on reflex responses. Curschmann noted that Hoffmann sign could be observed in some healthy or nervous individuals, and later authors have mentioned anxiety as a potential contributor [6, 7, 13, 16, 17]. However, empirical data on psychiatric symptoms among Hoffmann‐positive patients without cervical cord compression are limited.

The primary aim of this study was to describe the prevalence of anxiety and depressive symptoms in adults with a positive Hoffmann sign but no MRI evidence of cervical cord compression or myelomalacia. Secondary exploratory aims were to assess whether anxiety and depressive symptom categories differed by age group, sex, or history of psychiatric consultation.

## 2 Methods

This was a descriptive‐analytical cross‐sectional study conducted at the neurosurgery clinic of Beheshti Hospital, affiliated with Babol University of Medical Sciences. The study was designed to describe psychiatric symptom burden in a selected clinical population with a positive Hoffmann sign but no MRI evidence of cervical cord compression.

### 2.1 Participants and Recruitment

Participants were recruited consecutively from adult patients evaluated in the neurosurgery clinic who had a positive Hoffmann sign and underwent cervical spine MRI as part of clinical workup. Referral and MRI indication were determined by routine clinical judgment rather than by the study protocol alone. Therefore, the study population should be interpreted as a selected clinical cohort of Hoffmann‐positive patients referred for evaluation, not as a community sample and not as all patients with an isolated positive Hoffmann sign.

Eligible participants were adults aged 18 years or older with a positive Hoffmann sign and cervical MRI showing no significant cervical spinal cord compression and no T2 cord signal change. Hoffmann sign was assessed by a single neurosurgeon. The maneuver was performed by flicking the distal phalanx of the middle finger downward; involuntary flexion of the ipsilateral thumb and/or index finger was considered positive [6].

Patients were excluded if cervical MRI showed significant structural spinal cord compression or T2 signal change, or if they had a known movement disorder, previous cerebrovascular accident, cognitive impairment, or inability to cooperate with examination or questionnaire completion. Additional systematic investigations to exclude all possible non‐spinal neurological causes of upper motor neuron signs were not performed as part of the study protocol; this limitation is addressed in the Discussion.

### 2.2 Variables and Measurement

Demographic and clinical variables collected for this analysis included age group, sex, and history of psychiatric consultation. The demographic variable collected was biological sex; therefore, the term sex is used consistently throughout the manuscript.

Anxiety and depressive symptoms were assessed using the Hospital Anxiety and Depression Scale (HADS), a 14‐item self‐report questionnaire developed by Zigmond and Snaith [18]. The anxiety subscale (HADS‐A) and depression subscale (HADS‐D) each contain 7 items scored from 0 to 3, with subscale scores ranging from 0 to 21. Scores were categorized as normal (0–7), borderline [5, 12, 19], or clinically significant (> = 11) [20].

### 2.3 Statistical Analysis

Continuous variables are summarized as means and SDs. Categorical variables are summarized as counts and percentages, with numerators and denominators reported where possible. For key prevalence estimates, 95% CIs were calculated using the Wilson method. Associations between HADS categories and age group, sex, and history of psychiatric consultation were exploratory. Pearson *χ* ^2^ tests were used for categorical comparisons; when sparse cells were present, results were interpreted cautiously because of the limited sample size. All tests were 2‐sided, and *p* < 0.05 was considered statistically significant. *p* values are reported according to standard conventions. Analyses were performed using SPSS version 20.0 (IBM Corp., Armonk, NY, USA).

### 2.4 Ethics Statement

This study was approved by the Institutional Review Board of Babol University of Medical Sciences. All participants provided written informed consent before enrollment.

AI‐assisted editing: The final manuscript text was reviewed with ChatGPT (OpenAI, San Francisco, CA) solely to correct vocabulary and grammar. All AI‑suggested edits were verified and approved by the authors.

## 3 Results

### 3.1 Participant Characteristics

The study included 99 participants with a positive Hoffmann sign and no MRI evidence of cervical cord compression or T2 cord signal change. The cohort was predominantly female: 89 of 99 participants were female (89.9%; 95% CI, 82.4%–94.4%) and 10 of 99 were male (10.1%). Nine of 99 participants (9.1%) reported a history of psychiatric consultation. Age‐group data were available for 89 participants in the analytic tables.

### 3.2 Depression and Anxiety Scores

The mean HADS‐D score was 9.75 (SD, 2.96), and the mean HADS‐A score was 10.32 (SD, 2.74). Clinically significant depressive symptoms (HADS‐D > = 11) were present in 40 of 99 participants (40.4%; 95% CI, 31.3%–50.3%), borderline depressive symptoms in 40 of 99 (40.4%), and normal depressive symptom scores in 19 of 99 (19.2%). Clinically significant anxiety symptoms (HADS‐A > = 11) were present in 51 of 99 participants (51.5%; 95% CI, 41.8%–61.1%), borderline anxiety symptoms in 38 of 99 (38.4%), and normal anxiety symptom scores in 10 of 99 (10.1%).

### 3.3 Exploratory Associations

There were no statistically significant associations between sex or age group and HADS depression or anxiety categories in these exploratory analyses. The history of psychiatric consultation was associated with a higher depression category (*p *< 0.001) (Table 1)and higher anxiety category (*p *= 0.032) (Table 2). Because the number of male participants and the number with psychiatric consultation were small, subgroup findings should be considered descriptive rather than definitive.

**Table 1:** Comparison of depression categories by participant characteristics.

Variable		Depression status			*p* value
		Normal	Borderline	Depression	
Sex	Male	3/10 (30%)	3/10 (30%)	4/10 (40%)	0.659
	Female	16/89 (18%)	37/89 (41.6%)	36/89 (40.4%)	
Age	≥ 40	6/43 (14%)	17/43 (39.5%)	20/43 (46.5%)	0.210
	< 40	12/46 (26.1%)	17/46 (37%)	17/46 (37%)	
History of psychiatric disorder	Yes	0/9 (0%)	0/9 (0%)	9/9 (100%)	< 0.001
	No	19/90 (21.1%)	40/90 (44.4%)	31/90 (34.4%)	

*Note:* Age‐group data were available for 89 participants. HADS‐D categories: normal, 0–7; borderline, 8–10; clinically significant, > = 11. *p* values are from exploratory categorical comparisons.

**Table 2:** Comparison of anxiety categories by participant characteristics.

Variable		Anxiety status			*p* value
		Normal	Borderline	Anxiety	
Sex	Male	2 (20%)	4 (40%)	4 (40%)	0.322
	Female	8 (9%)	34 (38.2%)	47 (52.8%)	
Age	≥ 40	4 (9.3%)	11 (25.6%)	28 (65.1%)	0.199
	< 40	4 (8.7%)	21 (45.7%)	21 (45.7%)	
History of psychiatric disorder	Yes	0	1 (11.1%)	8 (88.9%)	0.032
	No	10 (11.1%)	37 (41.1%)	43 (47.8%)	

*Note:* Age‐group data were available for 89 participants. HADS‐A categories: normal, 0–7; borderline, 8–10; clinically significant, > = 11. *p* values are from exploratory categorical comparisons.

## 4 Discussion

In this cross‐sectional study of 99 Hoffmann‐positive patients without MRI evidence of cervical cord compression, anxiety and depressive symptoms were common. Clinically significant anxiety symptoms were present in 51.5% of participants, and clinically significant depressive symptoms were present in 40.4%. These estimates are higher than commonly reported population prevalence estimates for anxiety and depressive disorders [21, 22]. Because this study lacked a Hoffmann‐negative control group and enrolled a selected clinical population, the findings should be interpreted as descriptive and hypothesis‐generating.

The results may help explain why the Hoffmann sign has variable diagnostic performance in the literature. Hoffmann sign is not a disease‐specific test; it may reflect corticospinal tract dysfunction, but it can also occur in individuals without cervical cord compression [3, 6, 12, 13, 19]. Anxiety, arousal, and increased reflex excitability have long been suspected as possible contributors to positive reflex signs, but few data have directly addressed this possibility in Hoffmann‐positive patients without radiological CM [6, 7, 13, 16, 17]. Our findings support the need for further controlled studies rather than proving that anxiety or depression causes the sign.

These data should not be used to reduce or withhold neurological evaluation when objective examination abnormalities are present. In clinical practice, a positive Hoffmann sign should be interpreted together with symptoms, other upper motor neuron signs, strength, sensation, gait, functional status, and imaging findings. Psychiatric symptoms may be one contextual factor, particularly when Hoffmann sign is isolated and imaging is negative, but they should not replace appropriate neurological assessment.

The highly skewed sex distribution is an important feature of this cohort. Female participants represented 89.9% of the sample. Previous studies have reported that Hoffmann sign and hyperreflexia may be more common in women, possibly reflecting differences in reflex excitability [23, 24]. The sex distribution may also reflect referral patterns, the clinical population served by the study clinic, or the types of symptoms that led to cervical MRI. Because only 10 male participants were included, sex‐based comparisons were underpowered and should not be overinterpreted.

The history of psychiatric consultation was associated with higher anxiety and depression categories, which is clinically plausible and supports the internal consistency of the HADS findings. In contrast, age group and sex were not significantly associated with anxiety or depression categories. Given the small subgroup sizes and missing age‐group data for some participants, these negative findings should be interpreted cautiously.

This study has several limitations. First, there was no Hoffmann‐negative control group and no control group with MRI‐confirmed CM. Therefore, this study cannot estimate the independent effect of anxiety or depression on Hoffmann sign, nor can it determine diagnostic accuracy. Second, the recruitment process may have introduced selection bias because all participants were patients seen in a neurosurgery clinic and referred for MRI as part of clinical care. Third, although patients with known movement disorders, previous cerebrovascular accident, cognitive impairment, inability to cooperate, and cervical cord compression were excluded, the study protocol did not include a standardized neurological workup to exclude all other causes of upper motor neuron signs. This is an important potential confound because psychiatric comorbidities may coexist with organic neurological disease.

Fourth, detailed clinical symptoms and physical examination findings, such as pain, weakness, spasticity, gait disturbance, Babinski sign, other deep tendon reflexes, and functional scales such as the modified Japanese Orthopaedic Association score were not systematically recorded [9]. Fifth, Hoffmann sign was assessed by a single neurosurgeon, which may have improved internal consistency but does not allow assessment of interobserver reliability. Finally, the sample size was modest, and subgroup analyses were exploratory.

Despite these limitations, the study addresses a relatively underexplored clinical question. The high prevalence of anxiety and depressive symptoms in this selected Hoffmann‐positive, MRI‐negative cohort suggests that future studies should include psychiatric symptom measurement, standardized neurological examination, clearly defined referral indications, and appropriate control groups.

## 5 Conclusion

Among adults with a positive Hoffmann sign and no MRI evidence of cervical cord compression in this selected neurosurgical cohort, anxiety and depressive symptoms were common. These findings suggest that psychiatric symptom burden may be relevant when interpreting an isolated positive Hoffmann sign, but they do not establish causality and should not be used to limit appropriate neurological evaluation. Larger controlled studies are needed to clarify whether anxiety or depression independently influences Hoffmann sign and whether incorporating psychiatric screening improves clinical interpretation.

## Author Contributions

**Ebrahim Hejazian:** conceptualization, project administration, supervision, funding acquisition, data curation, methodology. **Forouzan Sedaghat:** investigation, resources. **Sussan Moudi:** data curation, validation. **Hoda Shirafkan:** formal analysis, validation, software, data curation. **Hadi Ebrahimi:** investigation, methodology, supervision. **Hamed Ahmadian:** validation, formal analysis, supervision. **Amir‐Hosein Zohrevand:** investigation, resources. **Mostafa Esmaeilnia Shirvani:** investigation. **Morteza Sharifzadeh:** writing – original draft, data curation, investigation. **Amirreza Tahmasebzadeh:** writing – review and editing, visualization, software, resources, investigation, data curation.

## Funding

The authors have nothing to report.

## Conflicts of Interest

The authors declare no conflicts of interest.

## Transparency Statement

Ebrahim Hejazian affirms that this manuscript is an honest, accurate, and transparent account of the study being reported; that no important aspects of the study have been omitted; and that any discrepancies from the study as planned have been explained.

## Data Availability

The data that support the findings of this study are available from the corresponding author upon reasonable request.

## References

[1] M. G. Fehlings , L. A. Tetreault , K. D. Riew , et al., “A Clinical Practice Guideline for the Management of Patients With Degenerative Cervical Myelopathy,” supplement, Global Spine Journal 7, no. S3 (2017): 21S–27S. 10.1177/2192568217703088 PMC5684844 29164027

[2] J. A. Tracy and J. D. Bartleson , “Cervical Spondylotic Myelopathy,” Neurologist 16, no. 3 (2010): 176–187. 20445427 10.1097/NRL.0b013e3181da3a29

[3] A. Fogarty , C. O'Neill , N. Kumar , et al., “A Systematic Review of the Utility of the Hoffmann Sign for the Diagnosis of Degenerative Cervical Myelopathy,” Spine (Phila Pa 1976) 43, no. 23 (2018): 1664–1669. 29668564 10.1097/BRS.0000000000002697

[4] C. Cook , M. Roman , K. M. Stewart , et al., “Reliability and Diagnostic Accuracy of Clinical Special Tests for Myelopathy in Patients Seen for Cervical Dysfunction,” Journal of Orthopaedic and Sports Physical Therapy 39, no. 3 (2009): 172–178. 19252263 10.2519/jospt.2009.2938

[5] Y. Kato , M. Matsumoto , M. Nakamura , et al., “Accuracy and Reliability of Physical Signs as a Diagnostic Tool for Cervical Cord Compression: A Cross‐Sectional Study,” Spine Surgery and Related Research 9, no. 2 (2025): 157–163. 40223840 10.22603/ssrr.2024-0187 PMC11983121

[6] J. K. Houten and L. A. Noce , “Clinical Correlations of Cervical Myelopathy and the Hoffmann Sign,” Journal of Neurosurgery. Spine 9, no. 3 (2008): 237–242. 18928217 10.3171/SPI/2008/9/9/237

[7] H. Ü. Curschmann , “Ber Die Diagnostische Bedeutung Des Babinski'schen Phänomen Im Präurämischen Zustand,” Münchener medizinische Wochenschrift 58 (1911): 2054–2057.

[8] J. J. Denno and G. R. Meadows , “Early Diagnosis of Cervical Spondylotic Myelopathy: A Useful Clinical Sign,” Spine (Phila Pa 1976) 16, no. 12 (1991): 1353–1355. 1771463 10.1097/00007632-199112000-00001

[9] K. E. Misulis and T. C. Head , ed., Essentials of Clinical Neurophysiology, 3rd ed. (Elsevier Health Sciences, 2025).

[10] J. Chang , X. Li , Y. Zhao , et al., “Hoffmann's Sign in Cervical Spondylotic Myelopathy Patients: Pathological Insights From Neuroimaging,” Neurosurgical Focus 56, no. 6 (2024): E10. 10.3171/2024.3.FOCUS23837 38823056

[11] L. Smith , A. F. Khan , and Z. A. Smith , “Neuroimaging Assessment of Hoffmann's Sign in CSM Patients,” Neurosurgical Focus 58, no. 1 (2025): E10. 10.3171/2024.7.FOCUS24414 39742517

[12] E. H. Gruenberger , J. Smith , M. Lee , et al., “The Hoffmann Parallax: A Prospective Study to Determine the Benefit of Hoffmann's Sign,” Orthopedic Reviews (Pavia) 15 (2023): 77875. 10.52965/001c.77875 PMC10317513 37405273

[13] R. A. Grijalva , R. D. Patel , H. J. Kim , et al., “Hoffmann Sign: Clinical Correlation of Neurological Imaging Findings in the Cervical Spine and Brain,” Spine (Phila Pa 1976) 40, no. 7 (2015): 475–479. 25608244 10.1097/BRS.0000000000000794

[14] T. M. Annaswamy , S. M. Bierner , Y. Guan , et al., “Reliability and Repeatability of the Hoffmann Sign,” PM & R 4, no. 7 (2012): 498–503. 22543037 10.1016/j.pmrj.2012.02.019

[15] G. Shi , H. Tang , and J. Shan , “Effect of Wrist Joint Angle on the Examination of Hoffmann's Sign,” Journal of Clinical Neuroscience 89 (2021): 123–127.

[16] Brain, W. Russell Baron , Diseases of the Nervous System, 2nd ed. (Oxford University Press, 1940).

[17] H. G. J. Krouwer , P. E. Barkhaus , and P. G. Antuono , “The Reflexes of Hoffmann, Tromner, and Mayer,” In Neurological Eponyms, eds. P. J. Koehler , G. W. Bruyn , and J. M. S. Pearce (Oxford University Press, 2000), 127–135.

[18] R. P. Snaith and A. S. Zigmond , “Hospital Anxiety and Depression Scale (HADS),” In Handbook of Psychiatric Measures (American Psychiatric Association, 2000), 547–548.

[19] Z. Jiang , Y. Li , H. Wang , et al., “The Value of Clinical Signs in the Diagnosis of Degenerative Cervical Myelopathy: A Systematic Review and Meta‐Analysis,” Global Spine Journal 14, no. 4 (2024): 1369–1374. 37903098 10.1177/21925682231209869 PMC11289551

[20] I. Bjelland , A. A. Dahl , T. T. Haug , et al., “The Validity of the Hospital Anxiety and Depression Scale: An Updated Literature Review,” Journal of Psychosomatic Research 52, no. 2 (2002): 69–77. 11832252 10.1016/s0022-3999(01)00296-3

[21] Z. Steel , C. Marnane , C. Iranpour , et al., “The Global Prevalence of Common Mental Disorders: A Systematic Review and Meta‐Analysis 1980–2013,” International Journal of Epidemiology 43, no. 2 (2014): 476–493. 24648481 10.1093/ije/dyu038 PMC3997379

[22] G. Y. Lim , W. W. Tam , Y. Lu , et al., “Prevalence of Depression in the Community From 30 Countries Between 1994 and 2014,” Scientific Reports 8, no. 1 (2018): 2861. 29434331 10.1038/s41598-018-21243-x PMC5809481

[23] H. Chikuda , A. Seichi , K. Takeshita , et al., “Correlation Between Pyramidal Signs and the Severity of Cervical Myelopathy,” European Spine Journal 19, no. 10 (2010): 1684–1689. 20229121 10.1007/s00586-010-1364-3 PMC2989225

[24] J. A. Glaser , J. K. Curé , A. A. Bailey , et al., “Cervical Spinal Cord Compression and the Hoffman Sign,” Iowa Orthopaedic Journal 21 (2001): 49–52. 11813951 PMC1888193

